# Automated detection of cervical spondylotic myelopathy: harnessing the power of natural language processing

**DOI:** 10.3389/fnins.2025.1421792

**Published:** 2025-03-19

**Authors:** GuanRui Ren, PeiYang Wang, ZhiWei Wang, ZhiYang Xie, Lei Liu, YunTao Wang, XiaoTao Wu

**Affiliations:** ^1^Department of Orthopedics, Zhongda Hospital, Medical College, Southeast University, Nanjing, Jiangsu, China; ^2^Department of Spine Surgery, Zhongda Hospital, Medical College, Southeast University, Nanjing, Jiangsu, China; ^3^Xuyi County People's Hospital, Huai'an, Jiangsu, China

**Keywords:** Long Short Term Memory Network, machine learning, electronic health record, natural language processing, cervical spondylotic myelopathy

## Abstract

**Background:**

The objective of this study was to develop machine learning (ML) algorithms utilizing natural language processing (NLP) techniques for the automated detection of cervical spondylotic myelopathy (CSM) through the analysis of positive symptoms in free-text admission notes. This approach enables the timely identification and management of CSM, leading to optimal outcomes.

**Methods:**

The dataset consisted of 1,214 patients diagnosed with cervical diseases as their primary condition between June 2013 and June 2020. A random ratio of 7:3 was employed to partition the dataset into training and testing subsets. Two machine learning models, Extreme Gradient Boosting (XGBoost) and Bidirectional Long Short Term Memory Network (LSTM), were developed. The performance of these models was assessed using various metrics, including the Receiver Operating Characteristic (ROC) curve, Area Under the Curve (AUC), accuracy, precision, recall, and F1 score.

**Results:**

In the testing set, the LSTM achieved an AUC of 0.9025, an accuracy of 0.8740, a recall of 0.9560, an F1 score of 0.9122, and a precision of 0.8723. The LSTM model demonstrated superior clinical applicability compared to the XGBoost model, as evidenced by calibration curves and decision curve analysis.

**Conclusions:**

The timely identification of suspected CSM allows for prompt confirmation of diagnosis and treatment. The utilization of NLP algorithm demonstrated excellent discriminatory capabilities in identifying CSM based on positive symptoms in free-text admission notes complaint data. This study showcases the potential of a pre-diagnosis system in the field of spine.

## Introduction

Cervical Spondylotic Myelopathy (CSM) is recognized as the most prevalent cause of spinal cord dysfunction worldwide (Fehlings et al., 2013). Hospitalizations for CSM are projected to occur at a rate of 4.04 per 100,000 person-years, with a significant annual increase in the number of surgical procedures (Lad et al., 2009; Wu et al., 2013; Bakhsheshian et al., 2017). The timely identification and management of CSM are imperative in order to achieve optimal outcomes before the occurrence of spinal cord injury (Bakhsheshian et al., 2017). However, previous research has indicated a delay in diagnosis averaging 6.3 years, primarily attributed to the lack of specific complaints (Sadasivan et al., 1993). Consequently, there is a need to devise an automated pre-diagnosis tool that can expedite the time between symptom onset and treatment, thereby alleviating the labor-intensive manual efforts of surgeons.

The domain of natural language processing (NLP) empowers computers to analyze, comprehend, and utilize human language. This artificial intelligence (AI) technology has proven to be effective across various industries, particularly in the extraction of structured data from huge databases (Wyles et al., 2019). NLP is suitable for the medical sector, due to the prevalence of time-consuming clinical notes (Wyles et al., 2019). Prior research has demonstrated the benefits of NLP in enhancing risk stratification models and conducting computerized semantic analysis of clinical notes (Kreimeyer et al., 2017). In the context of investigating diagnosis and clinical decision-making under ambiguity, NLP demonstrated its utility in extracting clinically significant information from electronic health records (EHRs) to record initial diagnostic hypotheses (Jones et al., 2018; Castro et al., 2017; Afshar et al., 2019).

Unlike traditional neural networks, which often assume independence between inputs and outputs, such models fail to consider contextual information in sequential data such as text, audio, and video. Recurrent neural networks (RNNs) are deep learning algorithms that leverage continuous information across contexts. These networks consist of recurrent cells, which are influenced by both previous states and current input through feedback connections (Yu et al., 2019). In order to address the long-term dependencies encountered by conventional RNNs, the Long Short Term Memory Network (LSTM) cell incorporates a “gate” mechanism to enhance memory retention (Hochreiter and Schmidhuber, 1997). Previous studies have used machine learning to distinguish cervical spondylotic myelopathy from normal cases using X-rays and MRIs (Lee et al., 2022; Wang et al., 2018). Our research demonstrated that natural language-driven LSTM models can aid in diagnosing spinal disorders (Ren et al., 2022; Wang et al., 2024). However, timely access to medical imaging is limited in low-income countries. Developing a model based on patient complaints could reduce unnecessary visits and geographic barriers. Our objective is to use ML models based on NLP that can automatically identify CSM using the free-text complaints of patients.

## Materials and methods

### Data source

We enrolled patients diagnosed with cervical illness who received treatment at Zhongda Hospital Affiliated to Southeast University from June 2013 to June 2020. Patients with primary diagnoses in other organs or with insufficient clinical information were excluded from the study. A total of 1,214 Chinese free-text admission notes were examined, consisting of main symptoms, time of occurrence, and causes. The included cases consisted of 85 instances of cervical spondylotic radiculopathy (CSR), 19 cases of cervical tumors, 246 cases of cervical trauma, 12 cases of cervical infection, and 852 cases of cervical spondylotic myelopathy (CSM) (Table 1). Characteristic symptoms and signs of cervical spondylotic myelopathy (CSM) encompass various manifestations such as impaired manual dexterity, stiffness, proprioceptive loss, and diminished glove sensation in the hands. Positive symptoms were identified from free text describing the main symptom and reviewed by two researchers. Additionally, individuals may experience heightened urgency, frequency, or hesitation in urination, spasticity in the extremities, and gait dysfunction, including a stiff or spastic gait (Crandall and Batzdorf, 1966; Denno and Meadows, 1991). Two experienced surgeons independently confirmed each diagnosis through meticulous examination of hospital records, MRI scans, and CT scans.

**Table 1:** Distribution of cervical diseases.

	**Count**
CSR	85
Cervical tumors	19
Cervical trauma	246
Infection	12
CSM	852

CSR, Cervical spondylotic radiculopathy; CSM, Cervical spondylotic myelopathy.

### Data analysis

**Figure 1:**
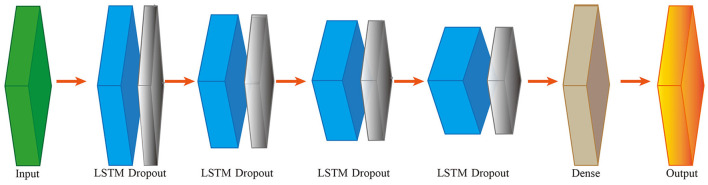
Inner structure of LSTM model. Bidirectional LSTM layers were conducted with dropout layers to inhibit overfitting. LSTM, Long Short Term Memory Network.

For data analysis, the Jieba package was employed in Python (version 3.7.6) to extract a word set comprising 428 words from the admission notes. The dataset underwent word set vectorization and was subsequently divided into a training set and a testing set at a random ratio of 7:3. Two NLP-based ML models were developed using the training set. Bidirectional LSTM models were implemented using the keras package in Tensorflow (version 2.3), with the addition of dropout for preventing overfitting (Figure 1). Detailed information of the LSTM model is shown in Table 2. The extreme gradient boosting (XGBoost) models were deployed using the Sci-Kit Learn package in Python (version 3.7.6).

**Table 2:** The framework of the LSTM model.

**Layer**	**Description**	**Output shape**	**Parameters**
1	Embedding	(None,19,160)	68,640
2	Bidirectional	(None,19,128)	115,200
3	Dropout	(None,19,128)	0
4	Bidirectional	(None,19,176)	152,768
5	Dropout	(None,19,176)	0
6	Bidirectional	(None,19,208)	233,792
7	Dropout	(None,19,208)	0
8	Bidirectional	(None,252)	337,680
9	Dropout	(None,252)	0
10	Dense	(None,1)	253

The testing set was used to compare the performance of the two algorithms, utilizing the following measures: (1) Recall quantifies the proportion of accurately classified true positives. (2) Accuracy represents the percentage of accurate predictions among all predictions made. (3) Precision denotes the ratio of correct predictions within positive predictions. (4) The F1 score enables the calculation of the harmonic mean between recall and precision. (5) The utilization of the Receiver Operating Characteristic (ROC) curve demonstrates the trade-off between sensitivity and specificity. The area under the curve (AUC) is a measure of the probability that a classifier would assign a higher rank to a randomly selected positive instance compared to a randomly selected negative instance, when normalized units are employed (Ford et al., 2016). Calibration curves and decision curve analysis enhanced the model's clinical applicability. The Shapley additive interpretation (SHAP) based on XGBoost model identifying key predictive features.

## Results

**Figure 2:**
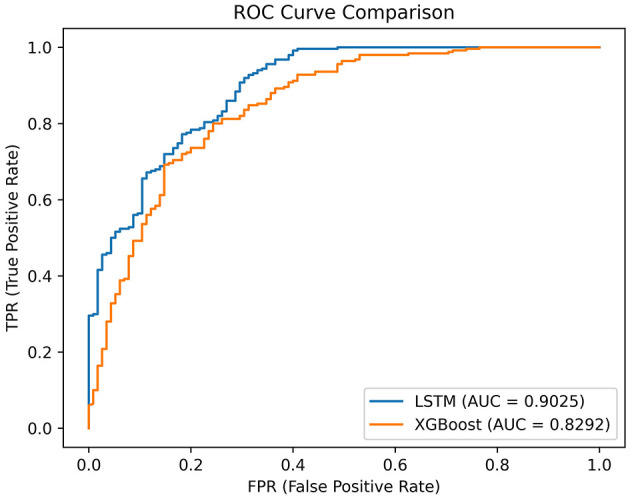
ROC curve of LSTM and XGBoost. LSTM, Long Short Term Memory Network; XGBoost, extreme gradient boosting.

**Figure 3:**
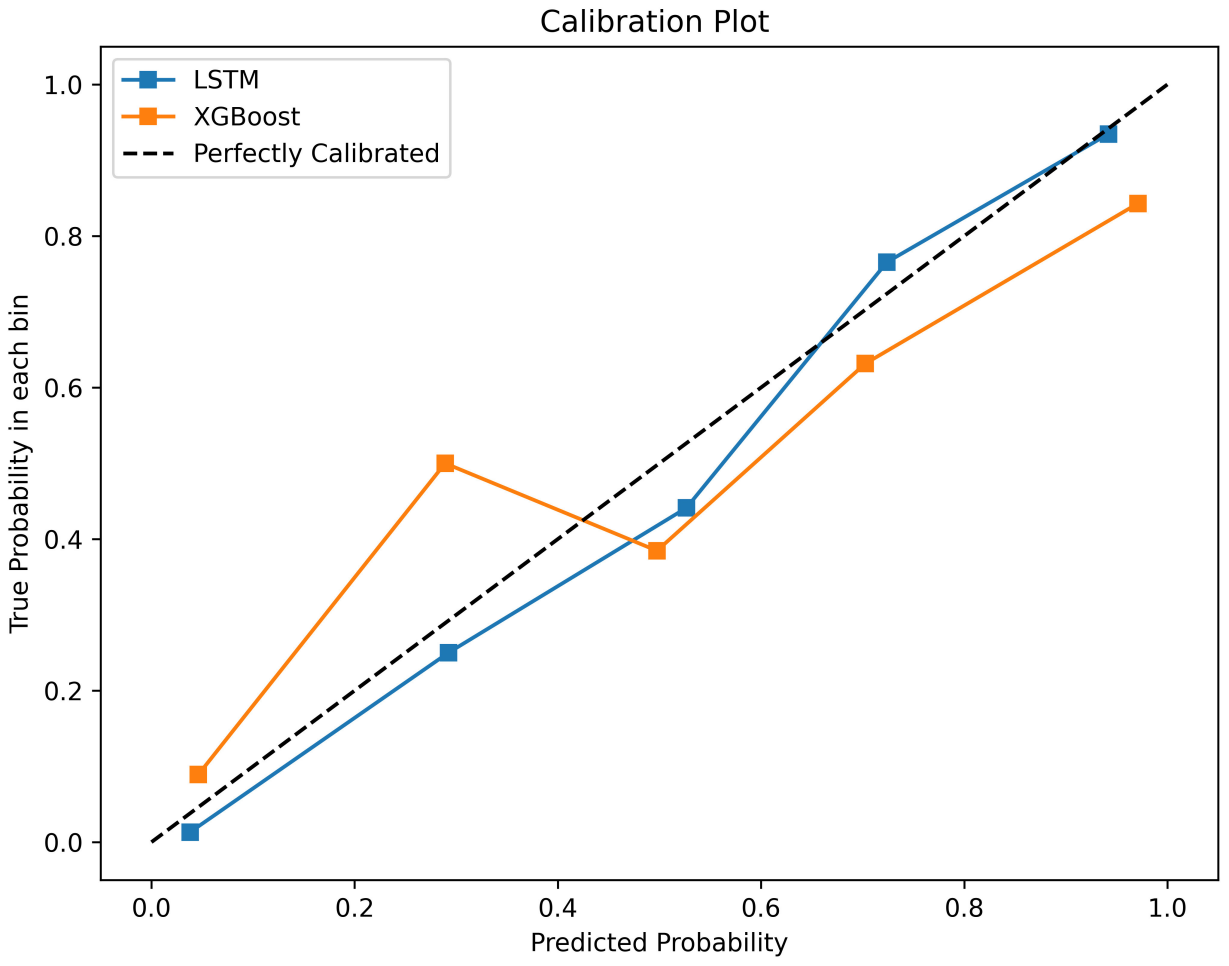
Calibration curves for LSTM and XGBoost.

**Figure 4:**
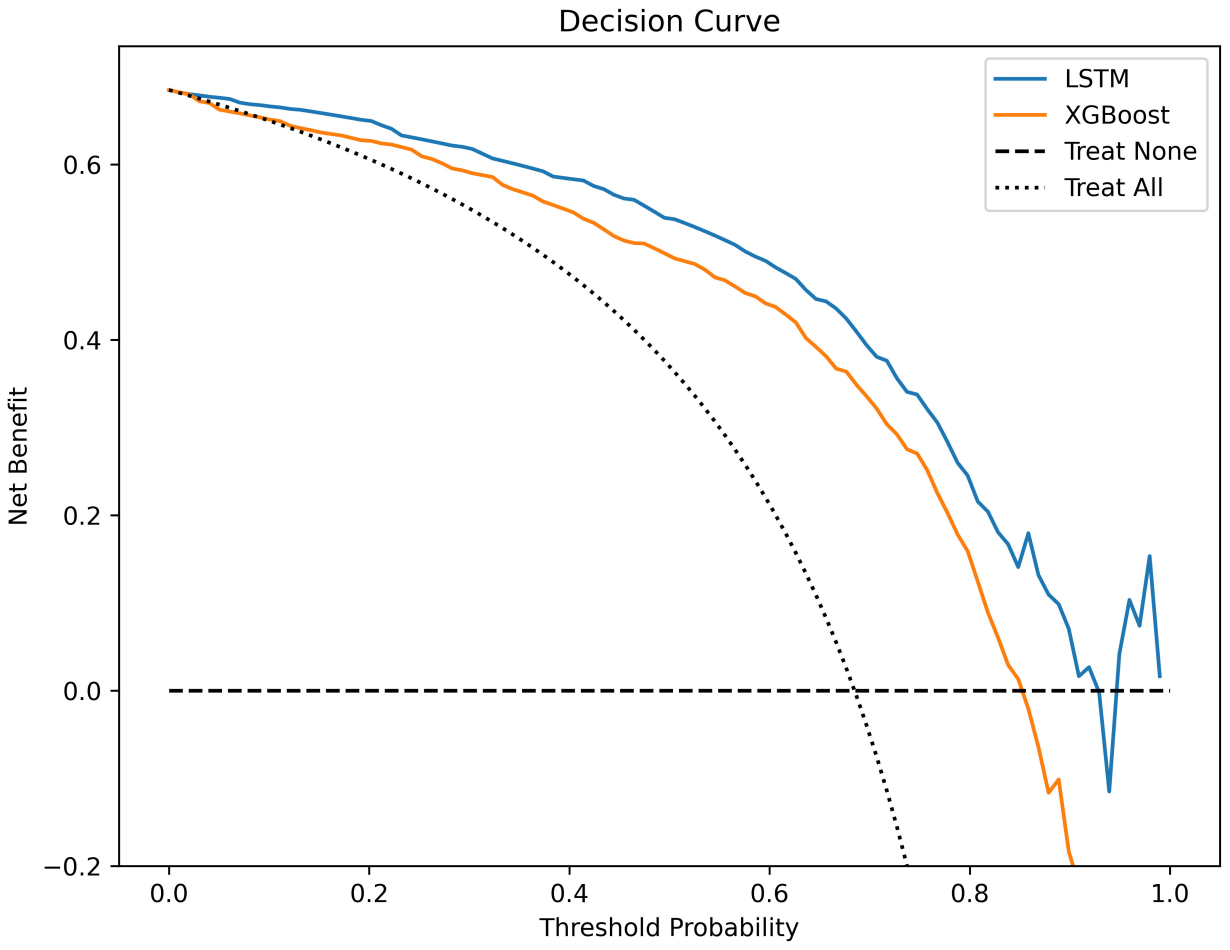
Decision curves for LSTM and XGBoost.

**Figure 5:**
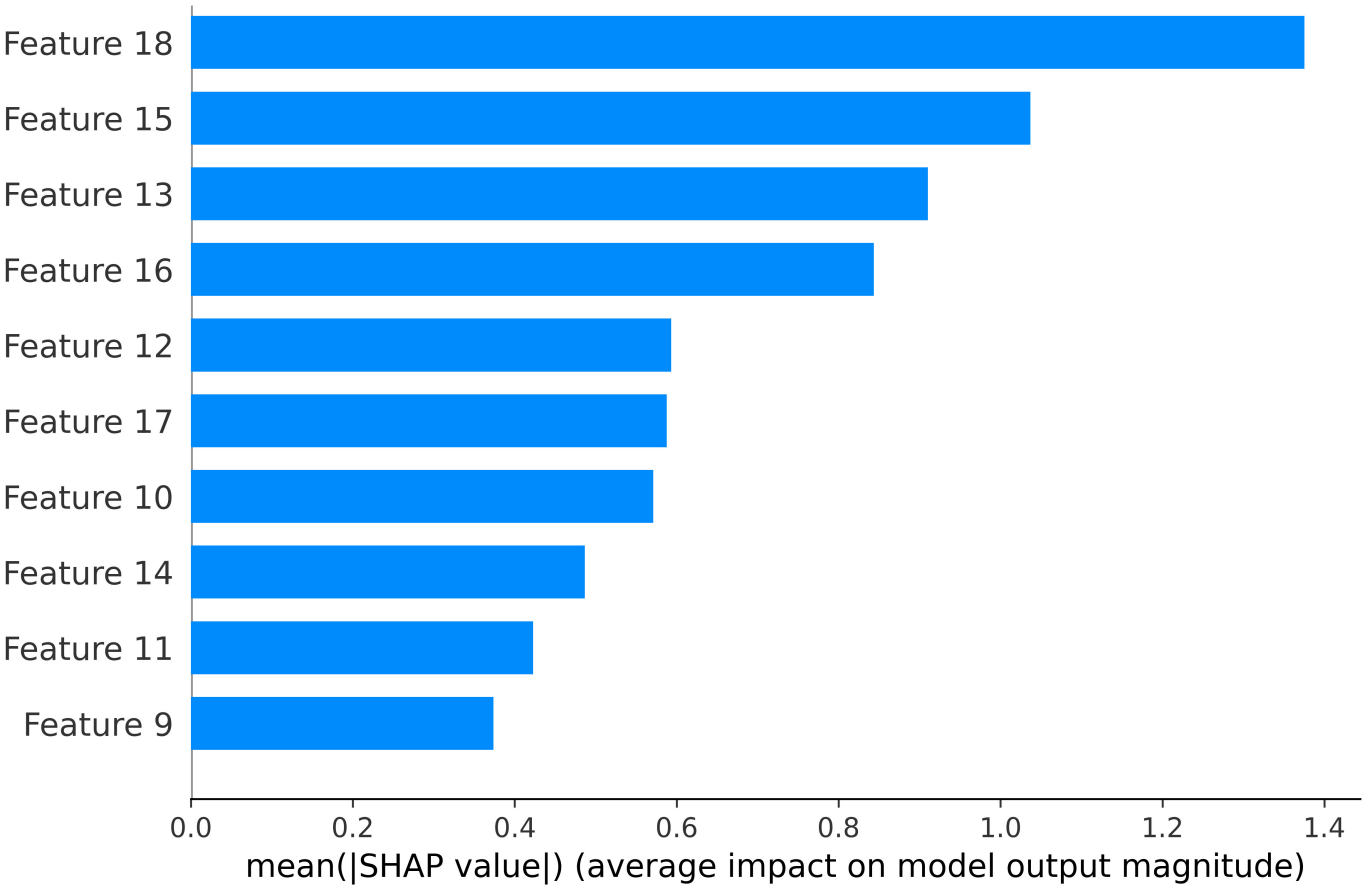
Feature importance based on SHAP.

In the test set, the LSTM model achieved an AUC of 0.9025 (Figure 2), an accuracy of 0.8740, a recall of 0.9560, an F1 score of 0.9122, and a precision of 0.8723 (Table 3). The XGBoost model obtained an AUC of 0.8292 (Figure 2), an accuracy of 0.8247, a recall of 0.9200, an F1 score of 0.8779, and a precision of 0.8394 (Table 3). The LSTM model demonstrated superior clinical applicability compared to the XGBoost model, as evidenced by calibration curves and decision curve analysis (Figures 3, 4). The SHAP based on XGBoost elucidated feature importance, highlighting the terms that describe the primary symptoms in the statement with high confidence. The most frequently occurring words include “numbness”, “weakness”, and “instability” (Figure 5).

**Table 3:** Performance of LSTM and XGBoost.

	**LSTM**	**XGBoost**
ACC	0.8740	0.8247
Recall	0.9560	0.9200
F1-score	0.9122	0.8779
Precision	0.8723	0.8394

LSTM, Long Short Term Memory Network; XGBoost, Extreme gradient boosting.

## Discussion

By utilizing positive symptoms in free-text admission notes that is easily accessible, the NLP-assisted tool has the capability to offer accurate medical guidance or conduct initial screening of cases. This study serves as a proof of concept for a spinal pre-diagnosis system, which has the potential to eliminate unnecessary visits and overcome geographical limitations (Zhou et al., 2021). The Medical pre-diagnosis system (Zhu et al., 2017) has garnered significant attention, with numerous disease prediction models being developed for neurological diseases (Güler and Ubeyli, 2007), pancreatic cancer (Wang et al., 2007), and diabetes (Barakat et al., 2010). The early identification of suspected CSM through the use of unstructured data has the potential to save patients time and reduce hospital costs, particularly in low-income countries with limited medical resources.

We developed NLP algorithms that effectively differentiate CSM based on positive symptoms in free-text admission notes. The LSTM model demonstrated accurate predictions for CSM, indicating the potential for predicting cervical illness using a limited amount of EHR data. The inclusion of cyclic connections allows LSTM to update its current state based on past states, making it suitable for analyzing sequential data (Yu et al., 2019). Due to its ability to handle long-term dependencies (Hochreiter and Schmidhuber, 1997), LSTM is widely used as an RNN model (Yu et al., 2019). XGBoost is a powerful model for predicting adverse events in free-text notes (Karhade et al., 2020a,b,c). However, Tassone et al. discovered that XGBoost exhibits lower performance compared to deep learning when dealing with big data (Tassone et al., 2020). While XGBoost achieves lower prediction accuracy than LSTM, this discrepancy may be attributed to the inherent difficulty of the current task, as both CSR and cervical tumors exhibit similar symptoms to CSM.

NLP algorithms leverage computer-based techniques to acquire, comprehend, and generate information in human language (Hirschberg and Manning, 2015). ML can enhance the efficiency of clinicians' workload, specifically for tasks that involve subjective judgment, thereby saving clinicians' valuable time (Reddy et al., 2019; Gambhir et al., 2016). Previous studies utilized NLP for automated surveillance of spine advent events (Karhade et al., 2020a,b,c, 2021). NLP predicted the severity of chest injuries using the initial eight hours of clinical records (Kulshrestha et al., 2021). An AI-based system diagnosed prevalent childhood diseases by extracting relevant information from EHRs using a hypothetico-deductive reasoning approach (Liang et al., 2019). Additionally, a Chinese medicine assistive diagnostic system was utilized to identify 187 well-known traditional Chinese medicine disorders and their associated symptoms, relying on unstructured freestyle records (Zhang et al., 2020). Furthermore, previous researches have predominantly concentrated on analyzing pathological changes through radiology reports (Huhdanpaa et al., 2018; Tan et al., 2018).

There are several limitations that necessitate acknowledgment. Firstly, this study was conducted retrospectively at a single hospital, which raises concerns about the generalizability of the findings. To ensure the broad applicability of the model and minimize potential bias, it is necessary to conduct external validation and employ prospective multi-institutional study designs. All patients included were surgical candidates, which inevitably enhances the severity of symptoms observed in our data compared to the overall population.

## Conclusions

Early identification of suspected CSM can facilitate timely confirmation of diagnosis and treatment. Our NLP algorithm exhibited commendable performance in diagnosing CSM through EHRs. The findings highlight the potential of a pre-diagnosis system that utilizes readily available descriptions of the primary symptom in the spine field.

## Data Availability

The original contributions presented in the study are included in the article/supplementary material, further inquiries can be directed to the corresponding authors.
